# Comparison of retention and fracture load of endocrowns made from zirconia and zirconium lithium silicate after aging: an in vitro study

**DOI:** 10.1186/s12903-022-02072-x

**Published:** 2022-02-16

**Authors:** Majid Sahebi, Safoura Ghodsi, Parsia Berahman, Amirhesam Amini, Somayeh Zeighami

**Affiliations:** 1 Dental Research Center, Dentistry Research Institute, Tehran University of Medical Sciences, Tehran, Iran; 2 Department of Prosthodontics, School of Dentistry, Tehran University of Medical Sciences, Tehran, Iran

**Keywords:** CAD-CAM, Compressive strength, Dental prosthesis retention, Endocrown, Zirconia

## Abstract

**Background:**

This study aimed to compare retention and fracture load in endocrowns made from translucent zirconia and zirconium lithium silicate.

**Methods:**

Fifty-six intact human maxillary molars after being mounted in acrylic resin, were scanned to acquire biogeneric copies. Specimens underwent standard endodontic treatment and were prepared for endocrown up to 2 mm above the cementoenamel junction. The specimens were randomly divided into two groups of 28, and endocrowns were designed using biogeneric copies and milled from high-translucent zirconia disks (Zr) and zirconium lithium silicate blocks (ZLS). After cementation with dual-cure resin cement, all the specimens underwent thermomechanical aging, and pull-out retention test and compressive test were conducted (14 specimens were used for each test in each group, n = 14), and failure modes in both tests were evaluated.

**Results:**

Independent samples t-test showed significant difference between the retention of Zr (271.5 N ± 114.31) and ZLS (654.67 N ± 223.17) groups (*p* value = 0.012). Compressive test results were also significantly different between Zr (7395.07 N ± 1947.42) and ZLS (1618.3 N ± 585) (*p* = 0.002). Failure mode of retention test was primarily adhesive failure at the cement-restoration interface in Zr group and cement-tooth interface in ZLS group. Failure modes of fracture test for Zr group were 7 non-restorable fractures and one restorable fracture while 6 specimens resisted compressive loads up to 8500 N without fracture. ZLS group showed 7 restorable and 7 non-restorable failures.

**Conclusions:**

Zr endocrowns showed significantly lower retention and higher fracture strength. Both materials seem to be suitable for fabrication of endocrown in clinical setup.

**Supplementary Information:**

The online version contains supplementary material available at 10.1186/s12903-022-02072-x.

## Background

There has always been the challenge of pointing out the best technique for restoring endodontically treated teeth [1]. The functional requirements and the extent of coronal tissue destruction are two most important determinants in selecting the most efficient way of restoring devitalized teeth; therefore, a single treatment plan cannot be applied to all the cases [2]. Endodontically treated teeth are prone to biomechanical failures [3]. The primary reason can be attributed to the reduction in stiffness and fracture resistance which are consequences of loss of integrity (caries, trauma and access cavity preparation) [4]. Using intracanal posts might seem essential due to the extensive loss of coronal tissue and lack of sufficient retention [5]. Although clinical success of post, core and crown has been ascertained in the literature, this method is not without shortcomings [3, 6].

The complications of routine procedures have led to the introduction of novel methods and restorations including endocrown, which was innovated in 1995 by Pissis [7]. These adhesive monoblock full ceramic restorations play the role of post, core and crown at the same time [5]. Bindl et al. reported survival rate of 87.1–94.6% after 55 months for molar teeth restored with this technique. Also, 10-year survival and success rate of these restorations were reported 99.0% and 89.9%, respectively by Belleflamme et al. [8, 9]. This category of restorations is mainly, but not exclusively, used in the molar region and benefits from both macro- and micro-retention [10–12]. As micro-retention plays the fundamental role in the retention of an endocrown restoration, it seems essential to use a material which can be etched and adhesively bonded; for example, glass containing ceramics like zirconium lithium silicate [10–13]. This newly introduced glass ceramic, besides from outstanding aesthetics, has mechanical properties similar and even higher than lithium disilicate [13]. On the other hand, zirconia, a polycrystalline ceramic with no amorphous glassy phase, has drawn lots of attention to its superb mechanical characteristics [14–16], especially as a material to be used in high-stress conditions [17, 18]. Zirconia cannot be etched by routine methods, and its retention in partial coverage restorations, like endocrown, is not as reliable as other ceramics. It is noteworthy to mention that debonding is the most common cause of failure in endocrown restorations [19, 20]. However, a combination of mechanical and chemical treatments has proved to be beneficial in increasing the bond strength of zirconia with tooth; for example, air-abrasion with alumina particles (combined with the use of chemical promoters like 10-MDP-based products) [21].

Few studies have assessed the mechanical behavior and clinical performance of zirconia endocrowns and have mostly focused on fracture strength [22–26], while other characteristics of these restorations including retention, adaptation and different designs are not studied adequately. The study conducted by Zou et al. reported no failures after 3-year follow-up of molars restored with endocrowns made of monolithic zirconia [22]. On the other hand, El-Ma'aita et al. showed 82.4% survival rate for zirconia endocrowns after two years, and all the failures were due to the debonding of restoration [26].

Although Competency of zirconia as a material in crown fabrication has been verified in the literature [27, 28], there are fundamental differences between crown and endocrown regarding preparation design, area covered by the restoration, finish line width, and shape of the restoration.

Considering the fact that debonding is one of the prevalent causes of endocrown failure[19, 20], existence of fundamental differences between bonding of zirconia and glass ceramics which casts doubt on the competency of zirconia as a material for endocrown, and scarcity of data on the retention of zirconia endocrowns in laboratory and clinical studies, this study aimed to assess and compare the retention and fracture load of CAD-CAM fabricated endocrowns made of translucent zirconia (Zr) and zirconium lithium silicate (ZLS) after thermomechanical aging and to evaluate failure modes after retention and fracture tests. The null hypotheses were that there are no significant differences in retention and fracture load between Zr and ZLS.

## Methods

The sample size was calculated based on Skalskyi et al. study using two-sample t-test power analysis option of PASS 11 software (NCSS, Kaysville, United States) considering α = 0.05 and β = 0.2 [23]. Fifty-six intact human maxillary molars, extracted for orthodontic or periodontic reasons, were collected as specimens. None of the specimens were extracted more than 2 months prior to the study. The specimens were stored in Hank’s balanced saline solution at room temperature, and the storage medium was replaced every two weeks [29]. All of the specimens were measured by a vernier caliper (IP67 Waterproof Digital Caliper; INSIZE, Suzhou, China); the teeth had to be 11 mm ± 1 in buccolingual, 10 mm ± 1 in mesiodistal, and 7.5 mm ± 1 in occlusogingival dimensions to enter the study.

**Fig. 1:**
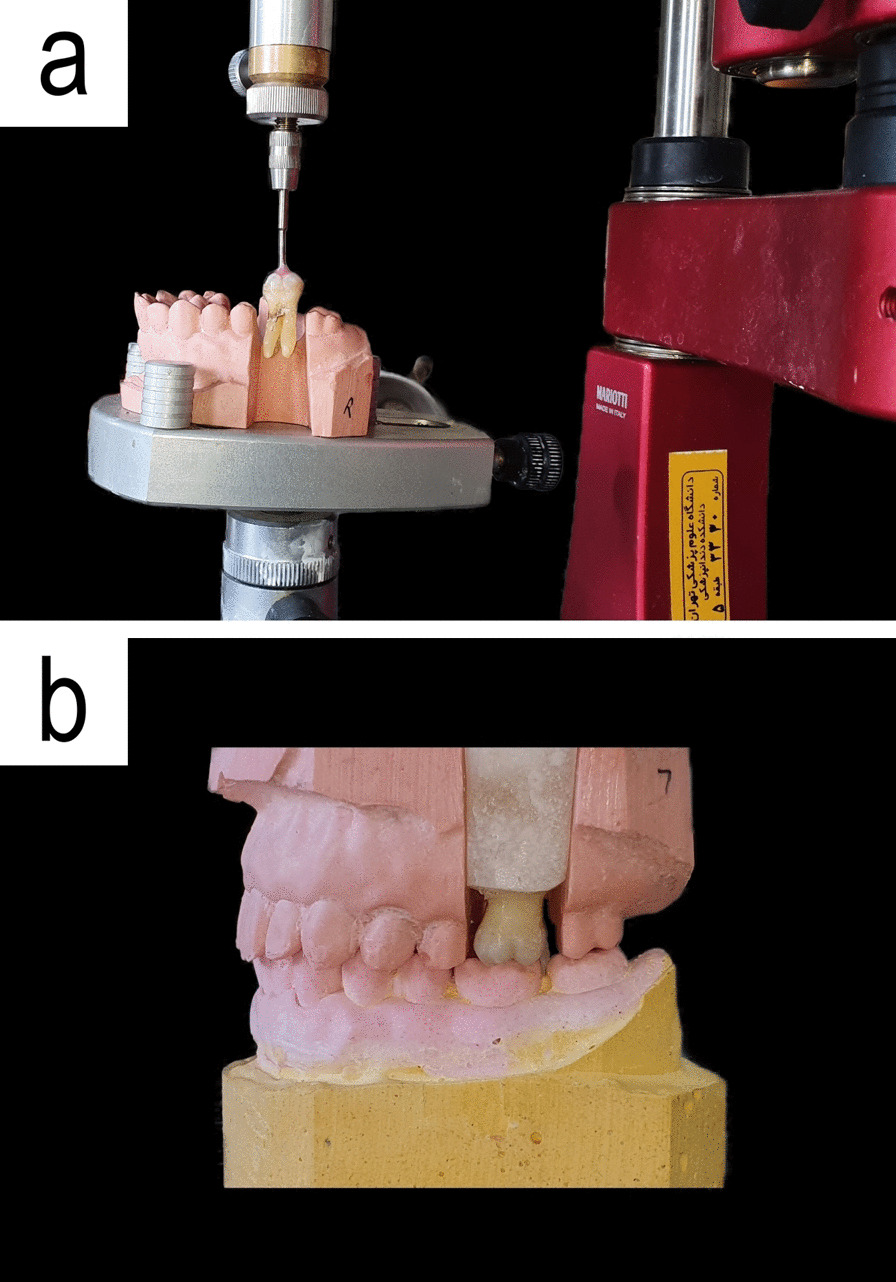
Mounting a tooth specimen using a surveyor (**a**) and final position of the tooth in occlusion with the opposite cast (**b**)

Maxillary and mandibular casts with perfect occlusion were made by pouring the impressions of a typodont (Prosthetic Restoration Jaw Model; Nissin, Kyoto, Japan) by type 4 dental stone (Asia chemi teb pharmaceutical Co, Tehran, Iran). A trapezoidal space was prepared on each side of the maxillary cast in the first molar area to be used as a mold for mounting the specimens. The teeth were mounted vertically by the aid of a surveyor (Cucciolo; Mariotti, Forli, Italy) in self-cure acrylic resin (Acropars 200; Marlic Dental, Tehran, Iran), and the mounted teeth were put in occlusion with the opposing cast (Fig. 1).

Before any intervention, the specimens were scanned by a laboratory scanner (inEos X5; Dentsply Sirona, York, United States) to acquire biogeneric copies to replicate tooth's preoperative anatomy. The teeth underwent standard root canal therapy and were prepared with 90° butt margin and 12° total occlusal divergence (6° for each axial wall) using a handpiece mounted on a surveyor [30, 31]. Specimens were reduced occlusally up to 2 mm above the cementoenamel junction. The teeth were inspected with a periodontal probe to have 4 mm pulp chamber depth; otherwise, they were replaced. Pulpal floor of the teeth with pulp chamber depths higher than 4 mm were lined with restorative glass ionomer (GC Fuji II; GC, Tokyo, Japan). The butt margin was polished and the pulp chamber was cleaned with 95% ethanol [31].

The specimens were divided into two groups of 28 by stratified random allocation, and the prepared teeth were scanned again. Endocrowns were designed using the biogeneric copies (inLAB CAD SW; Dentsply Sirona, York, United states). Cement space was set on zero for the margins and 50 µm for other areas [32]. The restorations were milled (inLab MC X5; Dentsply Sirona, York, United States) from high-translucent zirconia (DD Bio ZX^2^; Dental Direkt, Spenge, Germany) in Zr group and zirconium lithium silicate (Celtra Duo; Dentsply Sirona, York, United states) in ZLS group. Details of the two ceramics used in this study are summarized in Table 1 [33, 34]. The ZLS restorations were re-crystalized (at 820º C) and then glazed to reach the maximum mechanical properties [34], and the Zr restorations were fully sintered (at 1450 °C) and then glazed according to the manufacturer's instructions.

**Table 1:** Ceramic materials used in the study

Group	Manufacturer	Ceramic type	Composition	Flexural strength
ZLS (Celtra Duo)	Dentsply Sirona	Zirconium lithium silicate ceramic	SiO_2_ (58%), P_2_O_5_ (5%), Al_2_O_3_ (1.9%), Li_2_O (18.5%), ZrO_2_ (10.1%), Tb_4_O_7_ (1%), and CeO_2_ (2%)	Mill and polish: 210 MPa
				Mill and fire: 370 MPa
Zr (DD BIO ZX^2^)	Dental Direkt	High-translucent zirconium oxide ceramic	ZrO_2_ + HfO_2_ + Y_2_O_3_ (> 99%), Y_2_O_3_ (< 6%), Al_2_O_3_ (≤ 0.15%), and other oxides	1100–1250 MPa

Zr, zirconia; ZLS, zirconium lithium silicate

The seat of restorations was assessed using Vinyl Polyether Silicone (Fit checker; GC, Tokyo, Japan), and passive fit was confirmed by two impartial observers. Restorations in Zr group were prepared by air-abrasion with 50 µm alumina particles and cleaned using an ultrasonic device [35], while ZLS restorations were prepared using 9% hydrofluoric acid (Cera-Etch; Morvabon, Tehran, Iran) for 30 s and silane (Master-Dent Porcelain Primer; Dentonics, Charlotte, United States). The specimens were cemented by a dual-cure resin cement (Panavia F2.0; Kuraray, Tokyo, Japan) according to the manufacturer’s instructions. To standardize the cementation process, all the specimens were placed under 5 kg weight for 5 min. Cemented specimens were kept in an incubator at 37° for 24 h [36].

Specimens underwent thermomechanical aging including 5000 thermal cycles (C-300; Vafaei industrial factory, Qom, Iran) in 5 ° C and 55 °C distilled water with 25 s dwell time to simulate 6 months of clinical service [37, 38], and 500,000 loading cycles with 50 N force and 1.64 Hz frequency via a round metal cone (4 mm diameter) on central fossa of the restorations in an environment with 100% humidity (Chewing Simulator CS-4; SD Mechatronik, Feldkirchen-Westerham, Germany) to simulate 2 years of clinical service [38, 39].

**Fig. 2:**
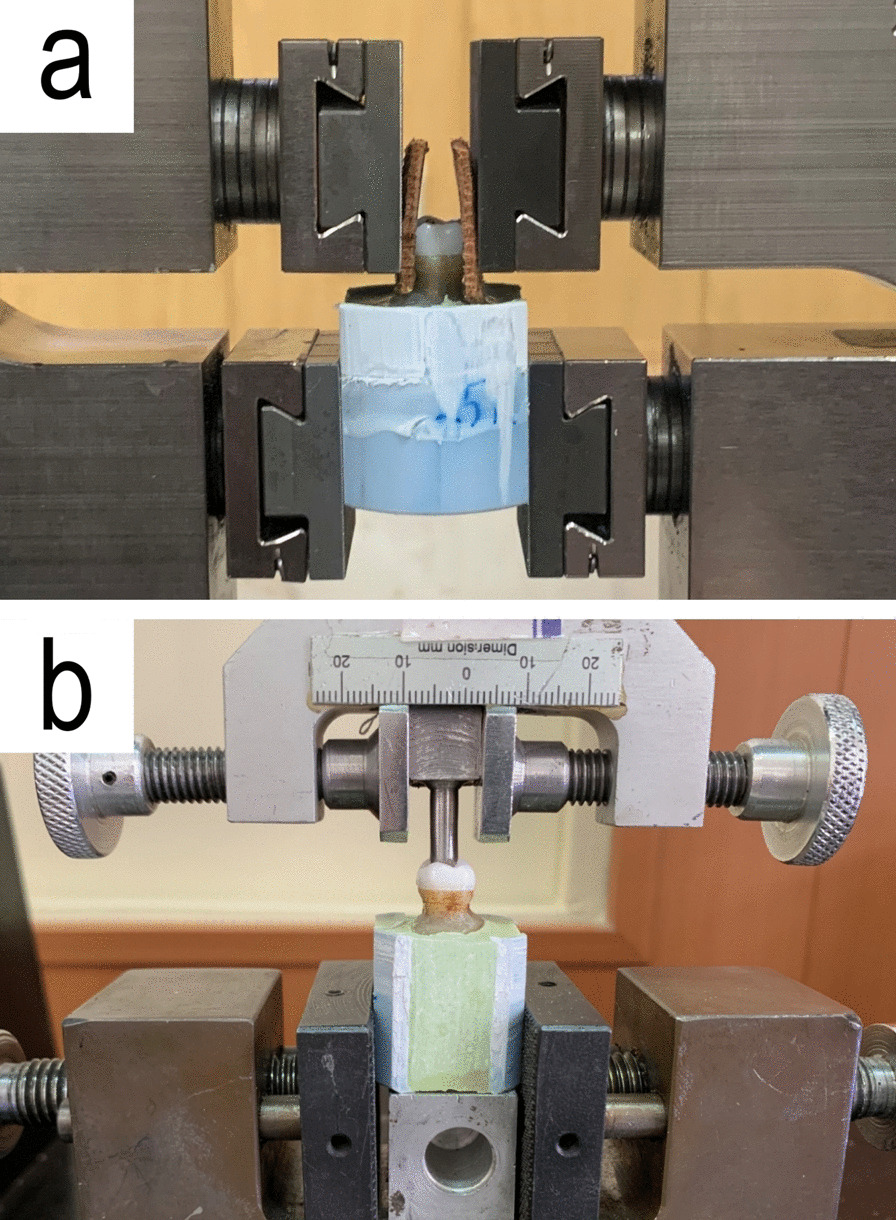
Specimen mounted in UTM during pull-out test (**a**) and compressive test (**b**)

14 Zr and 14 ZLS specimens were randomly selected for retention test. Each specimen was mounted individually in the lower compartment of universal testing machine (UTM) (ProLine; ZwickRoell, Ulm, Germany) while the endocrown part was held and fixed between two layers of leather and the upper compartment clips (Fig. 2a). The leather layers were used to reduce the possibility of endocrown fracture during tightening the upper compartment clips and to increase the friction between the two surfaces. UTM was set on pull-out mode with the speed of 5 mm/min, and the pull-out force was applied until complete separation of the restoration and the tooth [40].

**Fig. 3:**
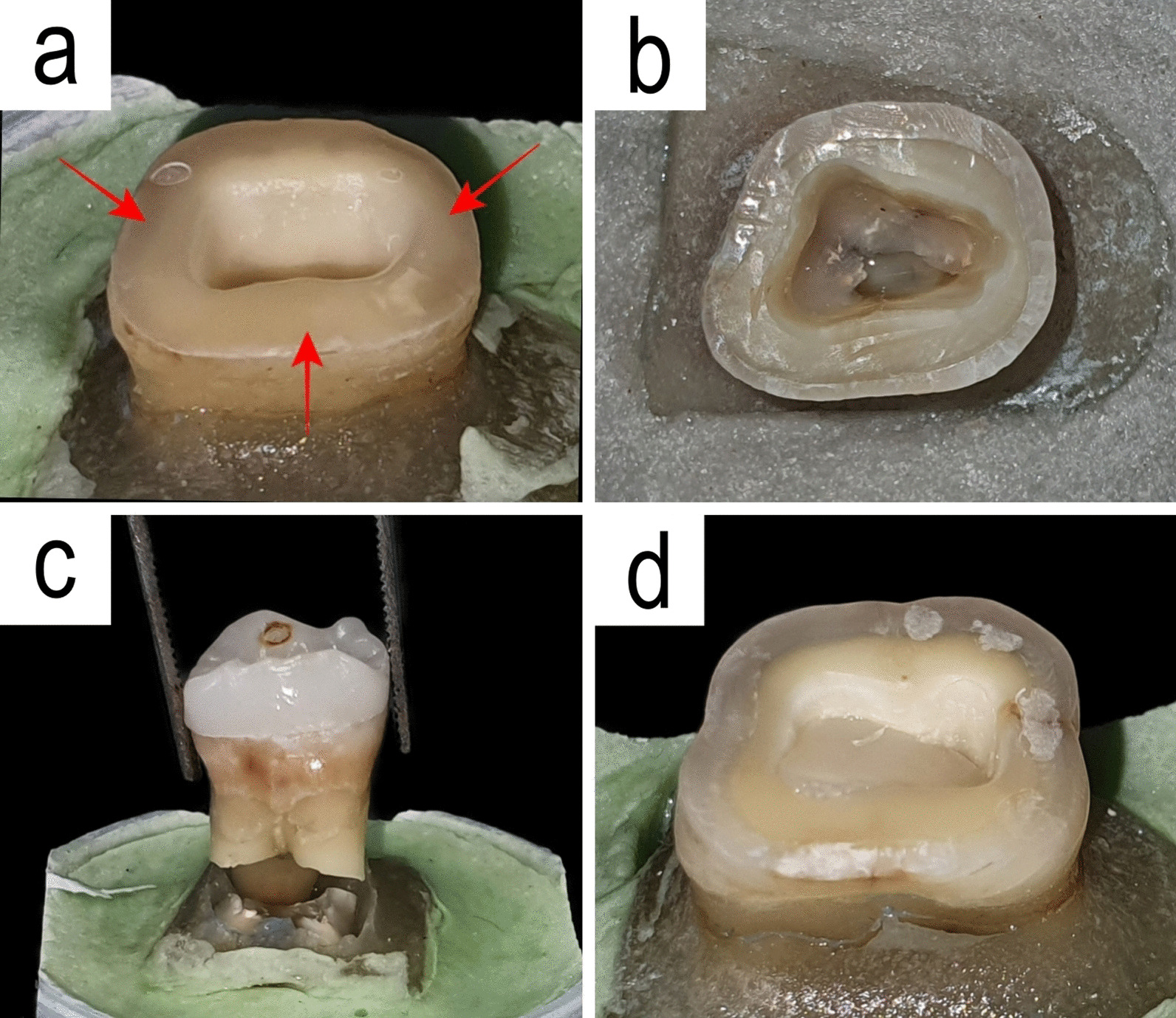
Specimens after retention test; **a** specimen in Zr group with type 3 failure and remaining cement (arrows) on the tooth structure, **b** specimen in ZLS group with type 2 failure and no remaining cement on the tooth structure, **c** specimen in ZLS group with fracture within the tooth (n = 1), **d** specimen in ZLS group with fracture in the pulp chamber part of restoration (n = 3)

Samples were assessed to evaluate failure mode after retention test (Fig. 3). Failure modes were classified as type 1 for cohesive failure of cement layer, type 2 for adhesive failure at the cement-tooth interface, type 3 for adhesive failure at the cement-restoration interface, and type 4 for mixed failures [41].

**Fig. 4:**
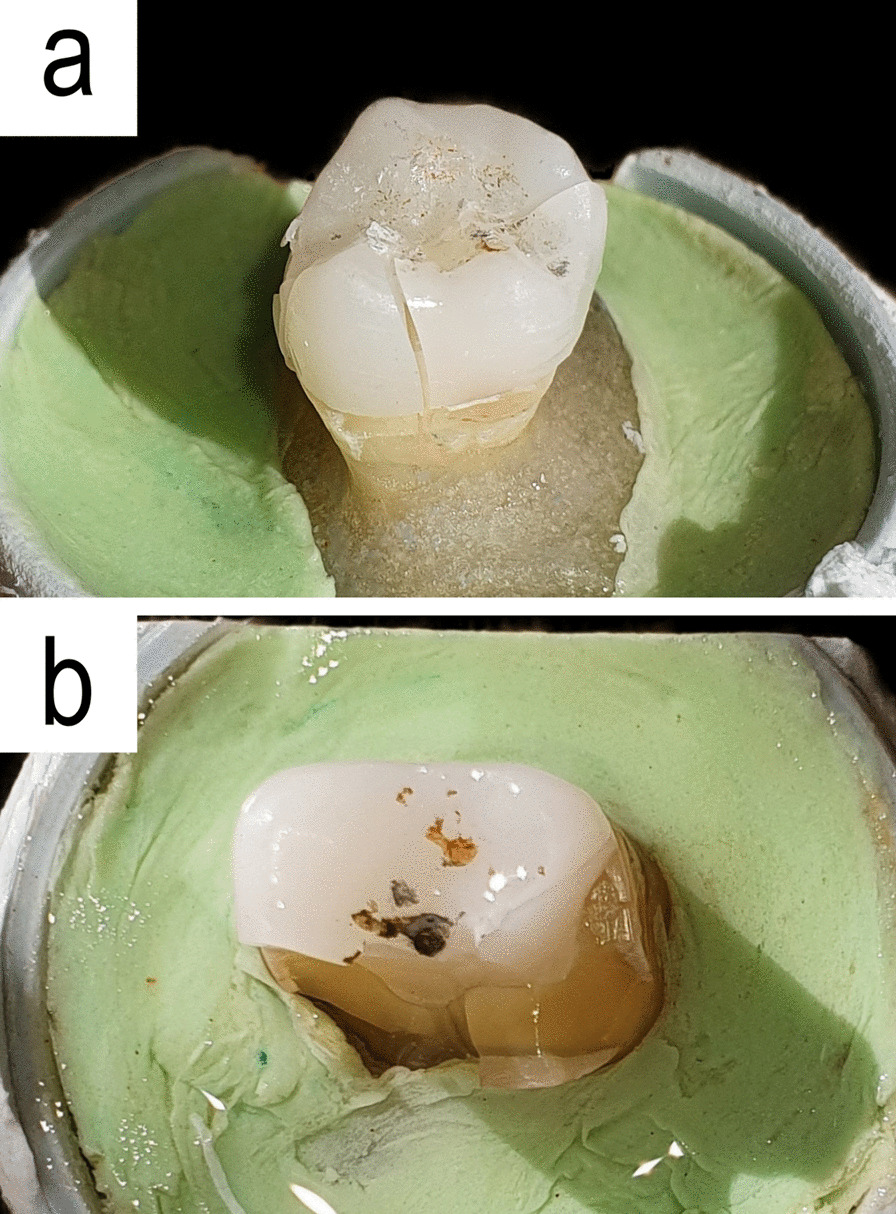
Specimen failure mode after compressive test; **a** type 1 failure (ZLS), **b** type 4 failure (Zr)

Compressive test was applied on the other 28 samples (14 Zr and 14 ZLS) with UTM and a 4 mm diameter metal cylinder with crosshead speed of 0.5 mm/min on the central fossa of the restorations until fracture or sudden fall in the load graph (Fig. 2b) [42]. All the specimens were evaluated for failure mode (Fig. 4). The failure modes of compressive test were classified as type 1 for cohesive failure in endocrown (restorable), type 2 for adhesive failure or fracture with displacement (restorable), type 3 for cohesive failure in enamel/dentine (non-restorable), and type 4 for cohesive failure in both tooth and ceramic structure (non-restorable) [43].

The data were analyzed using SPSS 22.0 (IBM, Armonk, United States). The normality of data in each group was evaluated using one-sample Kolmogorov–Smirnov test. The effect of material (ZLS vs. Zr) on retention and fracture load of endocrowns was assessed by Independent Samples t-test (α = 0.05).

## Results

Independent Samples t-test showed a significant difference between retention force of Zr group (271.50 N ± 114.31) and ZLS group (654.67 N ± 223.17) with *p* value of 0.012 (Table 2).

**Table 2:** Mean, standard deviation, minimum, and maximum of retention test results in Zr and ZLS groups

Group	Mean	Standard deviation	Minimum	Maximum	*P* value
Retention (N) (n = 14)					
Zr	271.50	114.31	104.71	490.22	0.012
ZLS	654.67	223.17	222.74	1006.25	

Zr, zirconia; ZLS, zirconium lithium silicate

All zirconia endocrowns were intact after retention test, and the retention failure mode was of type 3. Four of ZLS specimens fractured during the pull-out test; one endocrown was still bonded to the tooth while the tooth suffered root fracture, and the other 3 samples fractured from pulp chamber part of the endocrown. The other 10 samples in ZLS group suffered Type 2 failures.

Independent Samples t-test, also, showed that fracture loads in Zr group (7395.07 N ± 1947.42) were significantly higher than fracture loads in ZLS group (1618.30 N ± 585.00) with *p* value of 0.002 (Table 3).

**Table 3:** Mean, standard deviation, minimum, and maximum of fracture test results in Zr and ZLS groups

Group	Mean	Standard deviation	Minimum	Maximum	*P* value
Fracture load (N) (n = 14)					
Zr	7395.07	1947.42	4689.00	11,141.00	0.002
ZLS	1618.30	585.00	854.00	2752.00	

Zr, zirconia; ZLS, zirconium lithium silicate

Evaluation of the specimens after load-to-fracture indicated 7 type 4 failures (cohesive failure in both tooth and ceramic structure) in both Zr and ZLS groups which are considered non-restorable. The remainder of specimens in ZLS group (7 specimens) were subject of type 1 failure (cohesive failure in endocrown which is restorable). In the Zr group, however, one of the remaining specimens suffered type 1 failure, and the others remained intact up to 8500 N of compressive load (Table 4).

**Table 4:** Failure mode of fracture test in the two study groups

Both endocrown and tooth were intact (until 8500 N)		Restorable fracture	Non-restorable fracture
Failure mode of fracture test			
Zr	6	Type 1 (n = 1)	Type 4 (n = 7)
ZLS	–	Type 1 (n = 7)	Type 4 (n = 7)

Zr, zirconia; ZLS, zirconium lithium silicate

## Discussion

Fracture resistance along with esthetics and marginal adaptation are three of most important factors which determine the success of a restoration [18], and since debonding is the primary reason in failure of endocrown restorations [19, 20], this study was designed to assess the competency of translucent zirconia as a new material in fabricating endocrown restorations by comparing the fracture resistance and retention of these restorations with the ones made of a glass ceramic (ZLS).

To simulate the clinical setup as much as possible, human extracted teeth were used in the present study. The teeth were prepared with axial wall divergence of 6º (total wall divergence of 12°) as this degree of divergence has been used in previous studies, and it has been shown to balance the stress magnitude in the adhesive interface and lead to better adaptation of restoration by Tribst et al. and Darwish et al. [30, 44]. After preparation of the specimens for endocrown, the majority of teeth had 4 mm pulp chamber depth. For standardization of the specimens, the teeth with pulp chamber depths under 4 mm were excluded, and deeper pulp chambers were filled with a restorative material to reach 4 mm. 4 mm cavity depth is more than the minimum depth recommended by Fages et al. (3 mm) and has been used in other studies [31, 45, 46]. For the cementation of restorations, a dual-cure resin cement containing 10-MDP was used. The thickness of endocrowns and depth of bonding surfaces dictated the use of a cement with dual-curing mechanism to ensure complete polymerization; also, the presence of 10-MPD in the composition of the cement provided some degree of adhesive bond for the restorations made of zirconia.

To reproduce oral environment and engender fatigue in tooth-restoration complex and interfaces, the specimens underwent thermomechanical aging. In the present study, for retention test, the restorations were held using upper compartment of UTM and two layers of leather. There are ample studies on other types of restorations, crowns for example, which have used side or occlusal bars as handles to exert pull-out force on the restoration; however, there are very few studies on retention of endocrown restorations. In a pilot study, the methods previously mentioned, occlusal or side bars, could not overcome the bond strength of endocrowns made of ZLS, and the bars or extensions fractured before debonding of the restoration; therefore, the authors used this method to exert force directly from upper compartment of UTM to restorations.

The null hypotheses of this study were completely rejected since both fracture load and retention of endocrowns showed significant differences between Zr and ZLS groups.

Pull-out retention force in ZLS group was significantly higher than Zr group. Higher retention in ZLS endocrowns was expected as the glassy phase in ZLS structure allows the restorations in this group to be etched and adhesively bonded to the tooth structure [13]. On the other hand, restorations in Zr group did not benefit from the adhesive bond of resin cement as much as restorations in ZLS group. Sadighpour et al. showed 279.19 N ± 50.14 retention force in zirconia crowns prepared by air-abrasion with aluminum oxide particles and cemented with a dual-cure resin cement which is approximately similar to the retention force in Zr group in the present study (271.50 N ± 114.31) [47].

There is limited information on required retention force in molar restorations. Brunton et al. indicated that mean maximum opening force was 79 N in males with the maximum recorded opening force of 166.61 N [48]. The pull-out force which occurs in molar region while chewing sticky food has been recorded about 150 N [49]. Therefore, it can be concluded both zirconia and ZLS endocrowns have sufficient retention for restoring endodontically treated molars in clinical setup. Zou et al. assessed clinical performance of zirconia endocrowns and found no failures including debonding of restoration after 3 years [22], which seems to be an indication of sufficient retention at least in short term follow-ups.

Zirconia does not have the ability of being etched by routine methods [15, 16]. Air-abrasion with alumina particles, tribochemical conditioning, and other chemical and physical techniques have been proposed to improve the bond strength of zirconia to the tooth structure [15, 16]. The present study used air-abrasion technique along with cementation with a resin cement containing 10-MDP; however, this technique could not provide bond strength comparable to ZLS since the failure mode after retention test indicated that the majority of cement remained on restorations in ZLS group while in Zr group, cement was mostly remained on the tooth structure.

Zr showed fracture load significantly higher than ZLS group. This result was consistent with the results presented by other studies which showed Zr has higher fracture resistance compared to glass ceramics and even metal ceramic restorations [13, 25]. The mean maximum bite force in posterior region was recorded to be 738 N [50]. Both groups in the present study showed sufficient fracture resistances to be used in endocrown restorations in clinical cases. However, maximum voluntary bite forces up to 1642.8 N was recorded in Jansen van Vuuren et al. study which is higher than mean fracture load of ZLS group, but lower than Zr group [17]; therefore, it seems in high stress cases like bruxism, zirconia is a more reliable option to be used for endocrown fabrication.

Failure mode after fracture in ZLS group was divided into two groups of restorable and non-restorable with almost equal proportions while in Zr group, although the number of non-restorable cases were more than restorable cases, the fracture occurred in much greater forces than maximum bite force even in bruxer patients, and 6 specimens remained completely intact until compressive load of 8500 N.

The present study has the following limitations. Due to different study designs, interpretation of data in load-to-fracture tests are difficult, and presence of a control group including endodontically treated molars with maximum tissue preservation (class I composite restoration) could have helped the interpretation of data. Also, in the analysis of the retention test, this study relied only on retention force, and not the retention strength (retention force divided by surface area). Designing studies, considering the mentioned limitations, could provide more reliable results.

## Conclusions

Within the limitations of the present study, following conclusions were obtained:

1. Zirconia endocrowns had fracture loads higher than endocrowns made of zirconium lithium silicate while zirconium lithium silicate endocrowns showed superior retention.
2. Both zirconia and zirconium lithium silicate seem to be suitable materials for fabrication of endocrown in restoring endodontically treated molars with regard to fracture resistance and retention.

## Supplementary Information

**Additional file 1.** The raw data of the present study, including data acquired in Zr and ZLS goups during retention and compresive tests.

## Data Availability

The datasets used and/or analyzed during the study are included in Additional file 1: Raw data.

## Abbreviations

UTM: Universal testing machine
10-MDP: 10-Methacryloyloxydecyl dihydrogen phosphate

## References

[1] Robbins JW (2002). Restoration of the endodontically treated tooth. Dent Clin N Am.

[2] Faria AC, Rodrigues RC, de Almeida Antunes RP, de Mattos MG, Ribeiro RF (2011). Endodontically treated teeth: characteristics and considerations to restore them. J Prosthodont Res.

[3] Zarone F, Sorrentino R, Apicella D, Valentino B, Ferrari M, Aversa R (2006). Evaluation of the biomechanical behavior of maxillary central incisors restored by means of endocrowns compared to a natural tooth: a 3D static linear finite elements analysis. Dent Mater.

[4] Chang CY, Kuo JS, Lin YS, Chang YH (2009). Fracture resistance and failure modes of CEREC endo-crowns and conventional post and core-supported CEREC crowns. J Dent Sci.

[5] Sevimli G, Cengiz S, Oruc MS (2015). Endocrowns: review. J Istanb Univ Fac Dent.

[6] Dietschi D, Duc O, Krejci I, Sadan A (2008). Biomechanical considerations for the restoration of endodontically treated teeth: a systematic review of the literature, part II (evaluation of fatigue behavior, interfaces, and in vivo studies). Quintessence Int.

[7] Pissis P (1995). Fabrication of a metal-free ceramic restoration utilizing the monobloc technique. Pract Periodontics Aesthet Dent.

[8] Belleflamme MM, Geerts SO, Louwette MM, Grenade CF, Vanheusden AJ, Mainjot AK (2017). No post-no core approach to restore severely damaged posterior teeth: an up to 10-year retrospective study of documented endocrown cases. J Dent.

[9] Bindl A, Richter B, Mörmann WH (2005). Survival of ceramic computer-aided design/manufacturing crowns bonded to preparations with reduced macroretention geometry. Int J Prosthodont.

[10] Lander E, Dietschi D (2008). Endocrowns: a clinical report. Quintessence Int.

[11] Biacchi GR, Basting RT (2012). Comparison of fracture strength of endocrowns and glass fiber post-retained conventional crowns. Oper Dent.

[12] Veselinović V, Todorović A, Lisjak D, Lazić V (2008). Restoring endodontically treated teeth with all-ceramic endo-crowns: case report. Stomatol Glas Srb.

[13] Traini T, Sinjari B, Pascetta R, Serafini N, Perfetti G, Trisi P (2016). The zirconia-reinforced lithium silicate ceramic: lights and shadows of a new material. Dent Mater J.

[14] Zarone F, Sorrentino R, Vaccaro F, Traini T, Russo S, Ferrari M (2011). Acid etching surface treatment of feldspathic, alumina and zirconia ceramics: a micromorphological SEM analysis. Int Dent S Afr.

[15] Zarone F, Di Mauro MI, Ausiello P, Ruggiero G, Sorrentino R (2019). Current status on lithium disilicate and zirconia: a narrative review. BMC Oral Health.

[16] Pilo R, Dimitriadi M, Palaghia A, Eliades G (2018). Effect of tribochemical treatments and silane reactivity on resin bonding to zirconia. Dent Mater.

[17] Jansen van Vuuren L, Broadbent JM, Duncan WJ, Waddell JN (2020). Maximum voluntary bite force, occlusal contact points and associated stresses on posterior teeth. J R Soc N Z.

[18] Contrepois M, Soenen A, Bartala M, Laviole O (2013). Marginal adaptation of ceramic crowns: a systematic review. J Prosthet Dent.

[19] Bernhart J, Bräuning A, Altenburger MJ, Wrbas KT (2010). Cerec3D endocrowns–two-year clinical examination of CAD/CAM crowns for restoring endodontically treated molars. Int J Comput Dent.

[20] Thomas RM, Kelly A, Tagiyeva N, Kanagasingam S. Comparing endocrown restorations on permanent molars and premolars: a systematic review and meta-analysis. Br Dental J. 2020 (in press). 10.1038/s41415-020-2279-y 33184483

[21] Scaminaci Russo D, Cinelli F, Sarti C, Giachetti L (2019). Adhesion to zirconia: a systematic review of current conditioning methods and bonding materials. Dent J (Basel).

[22] Zou Y, Bai J, Xiang J (2018). Clinical performance of CAD/CAM-fabricated monolithic zirconia endocrowns on molars with extensive coronal loss of substance. Int J Comput Dent.

[23] Skalskyi V, Makeev V, Stankevych O, Pavlychko R (2018). Features of fracture of prosthetic tooth-endocrown constructions by means of acoustic emission analysis. Dent Mater.

[24] Dartora NR, Maurício Moris IC, Poole SF, Bacchi A, Sousa-Neto MD, Silva-Sousa YT (2021). Mechanical behavior of endocrowns fabricated with different CAD-CAM ceramic systems. J Prosthet Dent.

[25] Kanat-Ertürk B, Saridağ S, KÖSeler E, Helvacioğlu-Yiğit D, Avcu E, Yıldıran-Avcu Y. Fracture strengths of endocrown restorations fabricated with different preparation depths and CAD/CAM materials. Dent Mater J. 2018;37:2. 10.4012/dmj.2017-035 29311428

[26] El-Ma'aita A, Al-Rabab'ah M, Abu-Awwad M, Hattar S, Devlin H (2021). Endocrowns clinical performance and patient satisfaction: a randomized clinical trial of three monolithic ceramic restorations. J Prosthodont.

[27] Mikeli A, Walter MH, Rau SA, Raedel M, Raedel M. Three-year clinical performance of posterior monolithic zirconia single crowns. J Prosthetic Dent. 2021 (in press). 10.1016/j.prosdent.2021.03.004 33865558

[28] Soleimani F, Jalali H, Mostafavi AS, Zeighami S, Memarian M (2020). Retention and clinical performance of zirconia crowns: a comprehensive review. Int J Dent.

[29] Is Khinda V, Kaur G, Brar G, Kallar S, Khurana H (2017). Clinical and practical implications of storage media used for tooth avulsion. Int J Clin Pediatr Dent..

[30] Tribst JPM, Lo Giudice R, dos Santos AFC, Borges ALS, Silva-Concílio LR, Amaral M (2021). Lithium disilicate ceramic endocrown biomechanical response according to different pulp chamber extension angles and filling materials. Materials.

[31] Fages M, Bennasar B (2013). The endocrown: a different type of all-ceramic reconstruction for molars. J Can Dent Assoc.

[32] Pedrollo Lise D, Van Ende A, De Munck J, Umeda Suzuki TY, Cardoso Vieira LC, Van Meerbeek B (2017). Biomechanical behavior of endodontically treated premolars using different preparation designs and CAD/CAM materials. J Dent.

[33] Al-Zordk W, Saker S (2020). Impact of sintering procedure and clinical adjustment on color stability and translucency of translucent zirconia. J Prosthet Dent.

[34] Attaallah AM, Zayed EM, Dabees SM, Ashour YY, Fahmy AEE (2019). Comparison between biaxial flexural strength and microstructure of polished and glaze-fired specimens of zirconia lithium silicate glass ceramic. J dent mater tech..

[35] Haralur SB, Alamri AA, Alshehri SA, Alzahrani DS, Alfarsi M (2020). Influence of occlusal thickness and radicular extension on the fracture resistance of premolar endocrowns from different all-ceramic materials. Appl Sci.

[36] Kameli S, Khani F, Bahraminasab M, Ghorbani R, Abbas FM (2021). Bond strength and microleakage of different types of cements in stainless steel crown of primary molar teeth. Dent Res J (Isfahan)..

[37] Smith ED, Martin FE (1992). Microleakage of glass ionomer/composite resin restorations: a laboratory study, 1: the influence of glass ionomer cement. Aust Dent J.

[38] Gale MS, Darvell BW (1999). Thermal cycling procedures for laboratory testing of dental restorations. J Dent.

[39] Taha D, Spintzyk S, Sabet A, Wahsh M, Salah T (2018). Assessment of marginal adaptation and fracture resistance of endocrown restorations utilizing different machinable blocks subjected to thermomechanical aging. J Esthet Restor Dent.

[40] Eisa NS, Essam EA, Amin RA, Sharkawy EL, ZR. (2020). Fracture resistance and retention of three different endocrown materials. Al-Azhar dent j girls.

[41] Sadighpour L, Fazel A, Geramipanah F, Allahdadi M (2014). Effect of resin cement mixing method on the retention strength of a CAD/CAM zirconia crowns. J Indian Prosthodont Soc..

[42] Schriwer C, Gjerdet NR, Arola D, Øilo M (2021). The effect of preparation taper on the resistance to fracture of monolithic zirconia crowns. Dent Mater.

[43] Ghoul WE, Özcan M, Tribst JPM, Salameh Z (2020). Fracture resistance, failure mode and stress concentration in a modified endocrown design. Biomater Investig Dent.

[44] Darwish HA, Morsi TS, El Dimeery AG (2017). Internal fit of lithium disilicate and resin nano-ceramic endocrowns with different preparation designs. Futur Dent J.

[45] Hayes A, Duvall N, Wajdowicz M, Roberts H (2017). Effect of endocrown pulp chamber extension depth on molar fracture resistance. Oper Dent.

[46] Shin Y, Park S, Park JW, Kim KM, Park YB, Roh BD (2017). Evaluation of the marginal and internal discrepancies of CAD-CAM endocrowns with different cavity depths: an in vitro study. J Prosthet Dent.

[47] Sadighpour L, Geramipanah F, Fazel A, Allahdadi M, Kharazifard MJ (2018). Effect of selected luting agents on the retention of CAD/CAM zirconia crowns under cyclic environmental pressure. J Dent (Tehran).

[48] Brunton PA, Loch C, Waddell JN, Bodansky HJ, Hall R, Gray A (2018). Estimation of jaw-opening forces in adults. Orthod Craniofac Res.

[49] Kawata T, Yoda N, Kawaguchi T, Kuriyagawa T, Sasaki K (2007). Behaviours of three-dimensional compressive and tensile forces exerted on a tooth during function. J Oral Rehabil.

[50] Braun S, Bantleon HP, Hnat WP, Freudenthaler JW, Marcotte MR, Johnson BE (1995). A study of bite force, part 1: relationship to various physical characteristics. Angle Orthod.

